# Relationships between family functioning and HIV/AIDS-related preventive behaviors among young students with sexual experience

**DOI:** 10.3389/fpubh.2026.1741081

**Published:** 2026-02-04

**Authors:** Xinmei Yang, Yang Chen, Xiaojing Xu, Jinhua Jie, Hailin Zhuang

**Affiliations:** 1 School of Public Health and Health Management, Fujian Health College, Fuzhou, China; 2 Department of Hospital Quality Evaluation and Medical Record Management, The Third People’s Hospital of Chengdu, Chengdu, China; 3 Disease Control Department, Minhou County Center for Disease Control and Prevention, Fuzhou, China

**Keywords:** family functioning, HIV/AIDS, preventive behaviors, self-monitoring, young students

## Abstract

**Objectives:**

The prevalence of human immunodeficiency virus (HIV) remains a severe public health issue, especially among young people. To determine whether family functioning and self-monitoring influence HIV/acquired immunodeficiency syndrome (AIDS)-related preventive behaviors among young students with sexual experience, we conducted a cross-sectional exploratory study.

**Methods:**

This study, which used a convenience sampling method, was conducted in Fuzhou, China. The questionnaires used for this research included the Adaptation–Partnership–Growth–Affection–Resolve (APGAR) scale, the Self-Monitoring Scale, and questions concerning HIV/AIDS-related preventive behaviors. Binary logistic regression, multiple linear regression, and the Karlson–Holm–Breen method were employed to assess the relationship between family functioning and HIV/AIDS-related preventive behavior and to identify the mediating role of self-monitoring in this context.

**Results:**

A total of 29,038 students participated in the survey, of whom 2,510 had sexual experience. Family functioning had positive effects on students’ attitudes toward HIV testing [odds ratio (OR): 1.763; *p* < 0.01], reading/hearing HIV/AIDS news (OR: 2.776; *p* < 0.001), seeking online information (OR: 2.815; *p* < 0.001), and engaging in discussions about HIV/AIDS with others (OR: 2.686; *p* < 0.001). Moreover, the results of the mediation analysis revealed that self-monitoring played a mediating role in the relationship between family functioning and HIV/AIDS-related preventive behaviors.

**Conclusion:**

Family functioning was mediated by self-monitoring and indirectly affected HIV/AIDS-related preventive behaviors. It is essential to promote family functioning education in public health programs and to develop family intervention tools. Practical activities pertaining to the prevention of HIV/AIDS should be added to the self-management content of sex education courses for young students.

## 1 Introduction

Acquired immunodeficiency syndrome (AIDS) is a chronic progressive infectious disease caused by human immunodeficiency virus (HIV) (1). The World Health Organization (WHO) estimated that 40.8 million people were infected with HIV by the end of 2024 (2). In 2024, 370,000 young people aged 15 and to 24 were newly infected with HIV (3). This trend indicates that 183,000 new cases of HIV will be diagnosed in adolescents each year by 2030 (3). Therefore, HIV/AIDS remains a significant global public health issue.

Contemporary society is undergoing increasing liberalization in terms of sexual attitudes and behaviors. Young individuals from diverse cultural backgrounds and with varying sexual orientations now frequently interact and cohabit. They have broken free from the traditional restrictive oversight imposed by parental authorities (3, 4). With the widespread adoption of digital technologies and the proliferation of smartphone-based social platforms have significantly expanded, adolescents’ access to sexual information has been substantially expanded through multiple media channels. This technological makes it easier to identify potential intimate partners and establish connections, potentially lowering barriers to sexual exploration among student populations (5). Research has indicated that sexual contact is the predominant mode of HIV transmission among members of the collegiate population (6). Therefore, in recent years, school health education has been vigorously implemented, aiming to enhance young students’ awareness of AIDS, increase their knowledge about HIV infection prevention and elevate AIDS-related awareness (7). However, empirical data indicate that only 14.69% of sexually active college students underwent HIV screening within the previous 12-month period (8). Compared with their sexually inexperienced peers, sexually active young students are exposed to an elevated risk of HIV infection. This phenomenon has prompted us to focus on HIV/AIDS prevention behaviors among young students.

Family functioning is reflected mainly in family members’ capacity to develop effective conflict resolution strategies, maintain open communication patterns, and practice adaptive emotional regulation (9, 10). Optimal family functioning encompasses multiple interrelated dimensions, including active parental engagement, appropriate supervision of adolescent peer networks, and high-quality dialog between parents and their teenage children (11). Many scholars have studied the effects of family function on adolescents and young people’s health, providing evidence to support its association with various outcomes. For example, healthy family relationships are significantly associated with lower odds of cigarette smoking, binge drinking, and marijuana use (12); lower depressive symptoms (13); less severe problem behaviors (14); and physical activity and dietary intake (15). In a study of 746 Hispanic eighth-grade adolescents and their primary guardians, Cordova et al. reported significant correlations between discrepancies in parent-adolescent perception of family functioning and increased engagement in HIV-related risk behaviors among this demographic group (11). Seloilwe et al. (16) conducted in-depth qualitative interviews with 40 young people, 20 key informants and 40 parents, reporting that inadequate parental supervision and ineffective intergenerational communication were significantly correlated with higher incidence rates of HIV risk behaviors among adolescents. In a cross-sectional study involving 765 adolescents, Zakiei et al. (17) administered questionnaires and reported a significant positive correlation between optimal family functioning and heightened HIV/AIDS risk awareness among family members. Sun et al. interviewed 72 parent–adolescent pairs and suggested that good family functioning may improve adolescent health and reduce HIV risk (18). In summary, these studies suggest that family functioning may play an important role in enhancing awareness of HIV/AIDS prevention among young students.

The Stimulus–organism–response (SOR) theory was proposed by Mehrabian and Russell in 1974 (19), separates various variables into three categories (20). This theory assumes that the environment contains various stimuli (S), such as external or environmental shock. Stimuli cause personal changes, such as in an organism’s internal state (O), which subsequently influence its behavioral response (R). As a psychology theory, SOR model has been applied to explain health issues. For example, Yang et al. (21) used the SOR model to identify factors of health anxiety. Wut et al. (22) used the SOR perspective to study work from home challenges during the pandemic era in Hong Kong. Duong et al. (23) reported that the compulsive use of ChatGPT is a stimulus that triggers cognitive and affective (organism) changes in users that consequently contribute to sleep disturbance. Self-monitoring represents an individual’s capacity and propensity to regulate self-presentation behaviors to achieve desired social impressions (24, 25). This psychological attribute enhances self-awareness of behavioral patterns, which facilitating more effective modulation of both actions and cognitive processes (26). According to SOR theory, individuals are positively influenced by external environments such as family functioning, which may stimulate the development of self-monitoring capacities, thereby affecting their adoption of health behaviors. Some researchers have studied this topic. For example, Kurita et al. (26) surveyed 1,509 community-dwelling older adults and found that sharing records and feedback reports with family members may enhance the sustainability of self-monitoring practices. Thus, we propose a research hypothesis based on SOR theory that enables us to examine how family function influences students’ self-monitoring, which affecting their HIV/AIDS-related preventive behaviors.

In summary, previous studies have demonstrated that family function is associated with HIV/AIDS-related preventive behaviors. Additionally, self-monitoring has been identified as a factor affecting individual health. However, few studies investigated how self-monitoring mediates the relationship between family functioning and HIV/AIDS-related preventive behaviors among sexually experienced young students. This study analyzed the relationship between family functioning and HIV/AIDS-related preventive behaviors among young students with sexual experience in China. Meanwhile, we investigated the mediating effects of self-monitoring on the association between family functioning and HIV/AIDS-related preventive behaviors. This study highlights the necessity of considering the psychological and behavioral state of a particular family and individual in the HIV/AIDS-related preventive practices. Consequently, it contributes to providing theoretical support for the development and implementation of interventions strategies, in order to improve HIV/AIDS-related preventive behavior among young students, particularly those young students with sexual experience.

## 2 Materials and methods

### 2.1 Data source and sample

This study features a cross-sectional design. A convenience sampling method was used to conduct a sample survey among students from 6 colleges and 7 universities in Fuzhou, Fujian Province, from December 2024 to January 2025. The inclusion criteria were as follows: (1) agreement to complete the questionnaire and provide informed consent online; (2) enrollment as college/university students; and (3) the ability to read in Chinese and complete the questionnaire independently.

Existing studies report that 4.91–16.6% of Chinese students engage in sexual behaviors (1, 27). In light of these findings, we adopted a conservative estimate of 15.0% for the prevalence of sexual behaviors among college students in Fujian Province. In Equation 1, α was set to 0.05; $Z_{1-\alpha /2}$ represents the standard normal distribution bounded by an alpha of 0.05; and δ represents the permissible error, which was set to 0.1 p (8). A sample size of *n* = 2,177 was calculated. Thus, when a 10% drop-out rate was assumed, a minimum sample size of 2,395 participants was needed.


$$n={\left(\frac{Z_{1-\alpha /2}}{\delta }\right)}^{2}\times p\times (1-p)$$ (1)

Participants accessed the web-based questionnaire using a designated online platform,^1^ where they were first presented with detailed study information and a consent form. The introductory materials explicitly stated the voluntary nature of participation, assured participants that their responses were completely anonymous, and guaranteed data confidentiality. To ensure data integrity, each participant was assigned a distinct identification code within the survey system. Technical safeguards were implemented to prohibit multiple submissions from the same IP address, thereby preventing duplicate entries. The research protocol did not incorporate any compensatory measures for involvement. The digital platform automatically documented both the geographical origin and the time stamp of each completed questionnaire. A total of 31,486 students participated in the study. After incomplete and invalid surveys were eliminated, 29,038 valid questionnaires remained. The exclusion criteria included (1) participants who did not fall into the age range of 18–30 years; (2) individuals with missing data pertaining to key study variables (including outcomes, exposures, or adjusted covariates); and (3) individuals who did not report a history of sexual activity (28). Ultimately, a total of 2,510 students were included in the analyses.

### 2.2 Measurements

#### 2.2.1 Dependent variables

In accordance with previous studies (29, 30), the HIV/AIDS prevention behaviors measured in this context included attitudes toward HIV testing, the frequency of reading/hearing HIV/AIDS news, online information seeking, and discussions with others concerning HIV/AIDS.

In terms of attitudes toward HIV testing, respondents who engaged in risky behaviors and actively participated in HIV testing were coded as 1, while respondents who refused to take HIV tests after risky behaviors were coded as 0.

With respect to the frequency of reading/hearing HIV/AIDS news, online information seeking, and discussions about HIV/AIDS with others, the responses were measured on a 5-point Likert scale: 1 = never, 2 = seldom, 3 = sometimes, 4 = often, and 5 = always. We focused on the time frame of the past year. Scores less than or equal to 3 were recoded as “no” and assigned a value of 0. Scores of 4 or higher were recoded as “yes” and assigned a value of 1 (1).

#### 2.2.2 Independent variable

The Family Adaptation–Partnership–Growth–Affection–Resolve (APGAR) scale was employed to assess participants’ perceived family functioning on the basis of a standardized quantitative measure. This instrument includes five domains that are evaluated using a 3-point Likert-type response format (range: 0–2). On the basis of the established scoring criteria, the summed scores were interpreted as follows: 7–10 points reflected good family functioning, 4–6 points suggested mild to moderate functional impairment, and scores ≤3 indicated significant family dysfunction (31). For the analysis, we operationally defined family dysfunction as a composite APGAR score less than 7 (coded as 0). In contrast, a score of ≥7 was defined as “good family functioning” (code 1) (32). The Cronbach’s *α* coefficient for this scale in this study was 0.963. The KMO value of the questionnaire is 0.912.

#### 2.2.3 Mediator variable

Self-monitoring served as the mediator in this study and was measured using the Self-Monitoring Scale, a tool developed by Snyder and Gangestad (33) and revised by Wang et al. (34). The participants responded to each of the eight items on a five-point Likert-type scale, with response options ranging from 1 (strongly disagree) to 5 (strongly agree). Higher composite scores on this measure indicate greater levels of self-monitoring propensity. The Cronbach’s *α* coefficient for this scale was 0.959. The KMO value of the questionnaire is 0.954.

#### 2.2.4 Covariates

We controlled for students’ gender (0 for female, 1 for male), major (0 for humanities and social sciences, 1 for science and engineering, 2 for medicine), school type (0 for university, 1 for college) (35), hometown region (0 for urban/suburban, 1 for rural) (35), mother’s or father’s level of education (0 for primary school or below, 1 for middle school or high school, 2 for college or above), grade (0 for a low grade, 1 for a high grade) (36), and only child status (0 for no, 1 for yes) (35).

In light of the link between HIV/AIDS knowledge and preventive behaviors (37), we also controlled for HIV/AIDS knowledge. HIV/AIDS knowledge was assessed with an 8-item questionnaire developed by the National Center for AIDS/STD Control and Prevention, China CDC (38). The assessment instrument employed a response scale (yes/no/uncertain) for individual items, in which correct answers were assigned a value of 1 point. Participants who achieved a cumulative score of 6 points or higher met the predefined threshold for the “awareness” category (1).

### 2.3 Statistical analysis

The data analysis included five main steps. First, descriptive analyses were conducted to describe the characteristics of the participants using the mean [standard deviation (SD)] for continuous variables and percentages for categorical variables. Second, binary logistic regression was used to analyze the relationship between family functioning and preventive behaviors. Third, to determine whether self-monitoring mediated the association between family functioning and preventive behaviors, this study applied binary logistic regression and multiple linear regression. The primary regression models were as follows:


$$Y=\alpha_{i}+\beta_{i}X+\delta_{i}C+\varepsilon$$ (2)

$$Y=\alpha_{j}+\beta_{j}X+\gamma_{j}M+\delta_{j}C+\varepsilon$$ (3)

where Y is a dependent variable representing attitudes toward HIV testing, reading/hearing HIV/AIDS news, online information seeking, or discussions about HIV/AIDS with others; X represents family function; M represents self-monitoring; and C represents the controlled variables. Prior to the incorporation of the mediator, the regression model includes constant terms and coefficients for both the predictor and control variables, represented by $\alpha_{i}$ , $\beta_{i}$ , and $\delta_{i}$ , respectively (refer to Equation 2). Following the introduction of the mediating effects, the updated model parameters are denoted as $\alpha_{j}$ , $\beta_{j}$ , $\gamma_{j}$ , and $\delta_{j}$ for the constant term, independent variable, mediator, and controlled variables, respectively (see Equation 3). To assess potential multicollinearity issues, we conducted diagnostic examinations by computing the variance inflation factor (VIF). The results demonstrated acceptable levels of collinearity, and the VIF values were less than 10, thus indicating no substantial multicollinearity concerns among the explanatory variables.

Fourth, mediation analyses were performed by using the Karlson–Holm–Breen (KHB) decomposition method to assess potential indirect pathways (39). Furthermore, the observed association between family functioning and preventive behaviors may be subject to endogeneity concerns. Given that the participants in this study completed self-report questionnaires, the sampling method was nonrandomized and potentially susceptible to confounding variables, thereby introducing possible self-selection bias. To address this methodological limitation, propensity score matching (PSM) was implemented to assess the impact of self-monitoring behaviors (40). Statistical analyses were conducted using Stata 17.0 (StataCorp LLC, College Station, TX), with a threshold of *p* < 0.05 indicating statistical significance.

## 3 Results

### 3.1 Participant characteristics

Table 1 provides a brief overview of the participants, who were, in total 2,510 Generation Z students with sexual experience. The majority of these participants were males (60.64%), were in high grades (59.12%), were not the only children (71.08%), were university students (64.10%), and were from rural areas (56.85%).

**Table 1:** Descriptive statistics of the sample.

Variables	Total sample (*N* = 2,510)	Family dysfunction (*N* = 1884)	Good family functioning (*N* = 626)
Attitudes towards HIV testing, *N* (%)			
No	304 (12.11)	257 (13.64)	47 (7.51)
Yes	2,206 (87.89)	1,627 (86.36)	579 (92.49)
Frequency of reading/hearing HIV/AIDS news, *N* (%)			
Low	1920 (76.49)	1,535 (81.48)	385 (61.50)
High	590 (23.51)	349 (18.52)	241 (38.50)
Frequency of online information seeking about HIV/AIDS, *N* (%)			
Low	2064 (82.23)	1,628 (86.31)	438 (69.97)
High	446 (17.77)	258 (13.69)	188 (30.03)
Frequency of discussions with others concerning HIV/AID, *N* (%)			
Low	2073 (82.59)	1,627 (86.36)	446 (71.25)
High	437 (17.41)	257 (13.64)	180 (28.75)
Self-monitoring, mean (SD)	23.14 (7.76)	22.05 (6.92)	26.43 (9.13)
HIV/AIDS-related knowledge, *N* (%)			
Unaware	647 (25.78)	538 (28.56)	109 (17.41)
Aware	1863 (74.22)	1,346 (71.44)	517 (82.59)
Major, *N* (%)			
Humanities and social sciences	1,172 (46.69)	884 (46.92)	288 (46.01)
Science and engineering	919 (36.61)	699 (37.10)	220 (35.14)
Medicine	419 (16.69)	301 (15.98)	118 (18.85)
Grade, *N* (%)			
Low grades (1–2)	1,026 (40.88)	746 (40.61)	280 (50.80)
High grades (3–5)	1,484 (59.12)	1,138 (59.39)	346 (49.20)
Gender, *N* (%)			
Male	1,522 (60.64)	1,159 (61.52)	363 (57.99)
Female	988 (39.36)	725 (38.48)	263 (42.01)
School type, *N* (%)			
College	901 (35.90)	690 (36.62)	211 (33.71)
University	1,609 (64.10)	1,194 (63.38)	415 (66.29)
Hometown region, *N* (%)			
Rural	1,427 (56.85)	1,119 (59.39)	308 (49.20)
Urban/suburban	1,083 (43.15)	765 (40.61)	318 (50.80)
Mother’s education level, *N* (%)			
Primary school and below	807 (32.15)	630 (33.44)	177 (28.27)
Middle school or high school	1,217 (48.49)	905 (48.04)	312 (49.84)
College and above	486 (19.36)	349 (18.52)	137 (21.88)
Father’s education level, *N* (%)			
Primary school and below	557 (22.19)	443 (23.51)	114 (18.21)
Middle school or high school	1,465 (58.37)	1,093 (58.01)	372 (59.42)
College and above	488 (19.44)	348 (18.47)	140 (22.36)
Only children, *N* (%)			
No	1784 (71.08)	1,353 (71.82)	431 (68.85)
Yes	726 (28.92)	531 (28.18)	195 (31.15)

Additionally, 1884 (75.06%) students reported family dysfunction. Among these students, 1,159 were male, 59.39% were in high grades, and 63.38% were recruited from universities. Only 28.18% of these students were only children.

### 3.2 Results of the binary logistic regression analysis

The data presented in Table 2 indicate that family functioning is related to the preventive behaviors of students. After controlling for the variables, Models 1, 2, 3, and 4 demonstrated that family functioning was associated with attitudes toward HIV testing [odds ratio (OR): 1.763, *p* < 0.01], reading/hearing HIV/AIDS news (OR: 2.766, *p* < 0.001), seeking online information (OR: 2.815, *p* < 0.001), and engaging in discussions about HIV/AIDS with others (OR: 2.686, *p* < 0.001).

**Table 2:** Regression results for the family functioning and preventive behaviors.

Outcomes	OR	*p*-value	95%CI
Model 1 Attitudes towards HIV testing	1.763	0.001	1.266, 2.454
Model 2 Reading/hearing HIV/AIDS news	2.766	*p* < 0.001	2.255, 3.393
Model 3 Online information seeking	2.815	*p* < 0.001	2.253, 3.517
Model 4 Discussions with others concerning HIV/AID	2.686	*p* < 0.001	2.147, 3.361

Logistic regression analysis was conducted to examine the relationship between family functioning and preventive attitudes and behaviors, controlling for HIV/AIDS-related knowledge, major, grade, gender, school type, hometown region, mother’s education level, father’s education level, only children.

### 3.3 Analysis of the mediating effect

Before we controlled for the mediating variables, family functioning was significantly associated with preventive behaviors, as illustrated in Table 2. Model 9 (Table 3) also indicates that family functioning was significantly correlated with self-monitoring.

**Table 3:** Mediating effect regression results.

Variables	Model 5 attitudes towards HIV testing OR (95%CI)	Model 6 reading/hearing HIV/AIDS news OR (95%CI)	Model 7 online information seeking OR (95%CI)	Model 8 discussions with others concerning HIV/AID OR (95%CI)	Model 9 self-monitoring coefficients (SE)
Family functioning	2.527^***^	2.192^***^	2.110^***^	1.923^***^	4.231^***^
	(1.760, 3.628)	(1.769, 2.716)	(1.665, 2.675)	(1.511, 2.447)	(0.349)
Self-monitoring	0.948^***^	1.060^***^	1.067^***^	1.074^***^	—
	(0.931, 0.965)	(1.045, 1.075)	(1.051, 1.084)	(1.058, 1.091)	—
Constant	9.420^***^	0.025^***^	0.012^***^	0.012^***^	20.010^***^
	(5.153, 17.217)	(0.015, 0.043)	(0.007, 0.023)	(0.006, 0.022)	(0.611)

^*^p < 0.05, ^**^p < 0.01, ^***^p < 0.001.

These models were controlled for HIV/AIDS-related knowledge, major, grade, gender, school type, hometown region, mother’s education level, father’s education level, only children.

To validate the mediating role of self-monitoring in this context, we conducted additional analyses on the basis of the KHB decomposition method. As presented in Table 4, the analysis yielded statistically significant estimates for HIV testing behavior, with a total effect of 0.701, a direct effect of 0.927, and an indirect effect of −0.226. These results confirm that self-monitoring serves as a significant mediator in the relationship between family functioning and engagement in preventive behaviors.

**Table 4:** KHB test for mediating effect.

Effect	*β*	SE	*p*	95% CI		Mediation (%)
				Lower	Upper	
Family functioning—Self-monitoring—Attitudes towards HIV testing						
Total effect	0.701	0.174	<0.001	0.361	1.042	
Direct effect	0.927	0.184	<0.001	0.565	1.289	
Indirect effect	−0.226	0.042	<0.001	−0.309	−0.143	−32.24
Family functioning—Self-monitoring—Reading/hearing HIV/AIDS news						
Total effect	1.031	0.106	<0.001	0.823	1.239	
Direct effect	0.785	0.109	<0.001	0.570	0.999	
Indirect effect	0.246	0.036	<0.001	0.176	0.317	23.88
Family functioning—Self-monitoring—Online information seeking						
Total effect	1.022	0.116	<0.001	0.794	1.250	
Direct effect	0.747	0.121	<0.001	0.510	0.984	
Indirect effect	0.275	0.040	<0.001	0.197	0.353	26.91
Family functioning—Self-monitoring—Discussions with others concerning HIV/AID						
Total effect	0.957	0.118	<0.001	0.727	1.188	
Direct effect	0.654	0.123	<0.001	0.413	0.895	
Indirect effect	0.304	0.042	<0.001	0.222	0.386	31.73

After we controlled for the heterogeneity of the samples between the two groups, the effects of family functioning on preventive behavior remained significant (as shown in Table 5).

**Table 5:** Effect of family functioning on preventive behaviors based on PSM.

Dependent variable	Matching method	ATT	SE	*T* value
Attitudes towards HIV testing	*K*-nearest neighbor (*n* = 3)	0.072	0.020	3.57
	Caliper matching	0.064	0.016	3.99
	Kernel matching	0.064	0.016	3.98
Reading/hearing HIV/AIDS news	*K*-nearest neighbor (*n* = 3)	0.154	0.027	5.64
	Caliper matching	0.170	0.024	7.09
	Kernel matching	0.170	0.024	7.07
Online information seeking about HIV/AIDS	*K*-nearest neighbor (*n* = 3)	0.144	0.025	5.78
	Caliper matching	0.140	0.022	6.35
	Kernel matching	0.141	0.022	6.36
Discussions with others concerning HIV/AID	*K*-nearest neighbor (*n* = 3)	0.119	0.025	4.80
	Caliper matching	0.123	0.022	5.62
	Kernel matching	0.123	0.022	5.63

To validate the reliability of our estimates, we conducted a balance test across all matched samples. Postmatching analysis confirmed adequate covariate balance, with the standard deviation (SD) below the 5% threshold for all included covariates. PSM significantly reduced the difference after the balance test was satisfied (Table 6).

**Table 6:** Covariates balance testing for PSM.

Variable	Sample	Mean		Bias (%)	Reduct |bias| (%)	*T*-test	
		Treated	Control			*t*	*p* > *t*
Self-monitoring	U	26.43	22.05	54.0		12.59	<0.001
	M	26.19	25.91	3.4	93.7	0.61	0.54
HIV/AIDS-related knowledge	U	0.83	0.71	26.7		5.55	<0.001
	M	0.82	0.82	1.4	94.8	0.27	0.79
Major	U	1.73	1.69	5.1		1.11	0.27
	M	1.72	1.72	−0.7	85.4	−0.13	0.90
Grade	U	0.55	0.60	−10.4		−2.26	0.02
	M	0.56	0.55	3.2	69.7	0.55	0.58
Gender	U	0.58	0.62	−7.2		−1.57	0.12
	M	0.58	0.56	2.7	62.4	0.47	0.64
School type	U	0.34	0.37	−6.1		−1.32	0.19
	M	0.34	0.34	1.0	83.6	0.18	0.86
Hometown region	U	0.49	0.59	−20.6		−4.48	<0.001
	M	0.50	0.49	1.5	92.8	0.26	0.80
Mother’s education level	U	1.94	1.85	12.1		2.62	0.01
	M	1.93	1.95	−3.1	74.7	−0.53	0.59
Father’s education level	U	2.04	1.95	14.3		3.10	0.00
	M	2.04	2.04	−0.6	95.5	−0.11	0.91
Only children	U	0.31	0.28	6.5		1.42	0.16
	M	0.31	0.32	−2.3	64.2	−0.40	0.69

## 4 Discussion

This study revealed that among 29,038 students, only 2,510 reported having sexual experience, corresponding to 8.6%. Similar findings have been observed in other reports in Chinese university student populations. For example, Chen et al. (41) conducted a questionnaire among 4,892 freshmen at Huaqiao University and reported that the occurrence rate of sexual behavior among Chinese university students was 6.6%. A similar survey by Li et al. (42) revealed that 9.3% of students reported having sexual experience in Xuzhou. Chinese people usually hold relatively conservative attitudes toward sexuality, and the percentage of Chinese college students with sexual experience is lower than the commonly reported rates in Western countries. Madkour et al. (43) researched 15-year-olds from five Western countries and reported that the prevalence rate of sexual intercourse among this group ranged from 18.1 to 33.1%.

### 4.1 Family functioning and preventive behaviors

In this study, students with sexual experience had positive attitudes toward HIV testing, with more than 85% of students having active attitudes toward HIV testing if they engaged in risky behaviors. These results were similar to those reported by other Chinese scholars. For example, Ma et al. (44) conducted a study in a university in Eastern China and reported that 84.7% of the participants had a positive attitude toward the rapid detection of HIV. Our findings also confirmed that family functioning positively influenced students’ attitudes toward active HIV tests, which is similar to previous findings. He et al. (8) surveyed college students in the southwest of China, and the results revealed a significant association between parental communication patterns and HIV testing behaviors in sexually experienced college students. Specifically, students who received adequate sex education from their parents and engaged in more frequent discussions on sexual health matters demonstrated a higher participation rate of HIV testing. Several possible reasons may explain these findings. First, a good family environment helps reduce the stigma associated with HIV testing and assists family members in recognizing that seeking HIV testing is a normal and healthy behavior. Second, healthy family functioning emphasizes family members’ sense of responsibility for their own health and that of others, such as that of their partners and children. Active engagement in HIV testing could represent a concrete manifestation of this responsibility. Accordingly, through information exposure and communication with family members, individuals gain a clearer understanding of HIV-related risks and protective measures, as well as the importance of HIV testing. Third, family members may provide support for young students, such as assisting in locating testing facilities, accompanying family members to testing appointments, or interpreting the results of HIV test, thereby offering young students with greater support and a sense of security (45, 46). This type of family support not only reduces the pressure on young students during HIV testing, but also enhances their willingness and ability to take preventive actions. Overall, the family plays a positive role for its members. Through sound family communication and education, family members can serve as crucial resources for young students when facing health challenges, helping them make evidence-based decisions and taking actions to protect their own health and that of others. Therefore, to compensate for family dysfunction, society should take responsibility for implementing targeted interventions measures for high-risk groups. For students with weak family functioning, schools and communities should collaborate to provide supplementary psychological support and behavioral guidance. To increase HIV testing uptake, it is imperative to scale up service delivery through intensifying advocacy campaigns and promoting broader social engagement initiatives, targeting both the school population and the general public. Such efforts can help promote greater awareness of HIV-related risks and improve the understanding of the advantages associated with HIV testing (47). In addition, it is necessary to develop HIV prevention programs, especially those aimed at improving the accessibility, convenience and privacy of HIV testing services on campuses and elsewhere (8).

This study observed that family function is positively correlated with the frequency of reading/hearing HIV/AIDS news and searching for HIV/AIDS information on the internet, which is consistent with the findings of previous studies (48). Families are important for improving the health literacy of their members. Through daily communication and interaction, family members can share health knowledge and information with each other, which fostering a positive health environment. They can also collaboratively establish health goals and monitor each other’s progress of goal achievement (49). Research has also shown that family members can support other members at every stage of life in ways that cannot be replaced by other social subsystems. These findings indicate that the family has a strong effect on health promotion (50). In a family environment that prioritizes health education and information acquisition, family members are more inclined to engage in active health information seeking behaviors. Health knowledge should be regarded as a basic life skill, and family members are encouraged to proactively explore topics related to family health, which can increase their intrinsic motivation. Accordingly, they should actively utilize various information channels, such as authoritative health websites, information released by public health institutions, and news sources, to identify, search, evaluate, and integrate health-related knowledge. This approach can improve personal health literacy and decision-making ability. In addition, empirical evidence suggests that mass communication channels, including broadcast media, print journalism, and audio programs, serve as the predominant information sources for HIV/AIDS prevention among young people (51).

In a good family atmosphere, young people may be willing to discuss health-related topics, including HIV/AIDS, with other members of the family. Young people may also search for and collect information via online resources or news channels when they encounter confusing or interesting topics. College students are relatively important members of families and not only provide emotional support to middle-aged and older adults but also play a role in the dissemination of health information (52). Therefore, HIV/AIDS educational campaigns should preferably be conducted through mass media (especially social platforms such as public WeChat accounts, Weibo, and Douyin), in which context-aware messaging strategies should be designed for family-level interactions.

The study demonstrated a positive association between improved family functioning and increased engagement in HIV/AIDS-related discussions among participants. These findings are similar to those reported by Cordova et al. (11), and reflected the influence of family functioning on the degree of communication openness. Specifically, according to the McMaster Model of Family Functioning, the fundamental role of a family is to promote the physiological, psychological, and social health development of its members (53). Within a family system, communication serves as a basic mechanism to achieve the above functions, thereby promoting the development of both the family and its members. Therefore, young students with close family ties are more likely to possess the ability to initiate, listen to and address these important yet potentially difficult conversations. Moreover, as confirmed by our research, the family is an important environment for improving health literacy. Through daily communication and interaction, family members can share health knowledge and information with each other, fostering a positive health environment. They can also monitor each other’s health status. Research has demonstrated that young students effectively disseminate knowledge related to HIV/AIDS to their family members, which contributes to enhancing their understanding of HIV/AIDS and improving their attitudes toward these topics (54). When the topic of sexual health is discussed, trust and support within the family can reduce the associated sense of shame and stigma. This approach makes it easier for family members to talk about sensitive, private, and embarrassing topics, which promotes more open communication. Existing research has indicated that adolescents demonstrate a pronounced inclination to acquire initial knowledge regarding sensitive subjects through parental guidance (16). Consequently, young individuals prefer to obtain sexual health information primarily from family members pairs. The implementation of parental education programs could improve students’ levels of comfort, self-assurance, and understanding of reproductive health topics (16). In a healthy family environment, it is normal for family members to communicate with each other effectively and provide sufficient emotional support, psychological support, and resources to children. By perceiving positive emotional expression among family members, young people develop the ability to understand emotions and integrate these abilities into interactions with others. In daily interactions, they are more inclined to engage in helping and sharing while effectively avoiding problematic behaviors (55). Therefore, discussions about topics such as HIV/AIDS may occur among family members or extend beyond the family unit, such as between parents and children or among friends. Specifically, an open family atmosphere promotes members’ willingness and ability to engage in discussions on these topics.

### 4.2 The mediating role of self-monitoring

Individuals with exhibit high self-monitoring capacity are more sensitive to socioenvironmental stimuli and exhibit enhanced behavioral flexibility and improved regulation of self-presentation strategies (56). In addition, those who exhibit high self-monitoring capacity seem to be more adept at recognizing social cues (57), thus providing them with the key and necessary information to evaluate and determine their intended course of action. This study revealed that self-monitoring plays a negative mediating role in the relationship between family function and attitudes toward HIV testing. In a healthy family environment, the care, communication, and mutual attention among family members, although originally intended as support, may be interpreted by those with high self-monitoring as surveillance or judgment. They may perceive family members’ concern as “monitoring” their behaviors, especially those related to HIV-related risk, such as sexual activity and drug use. Thus, this attention may generate resistance. Feelings of being monitored increase individuals’ tendency to avoid HIV testing.

Self-monitoring plays a positive mediating role in the relationship between family functioning and reading/hearing HIV/AIDS news and online information seeking. This research revealed the mechanism how the family environment promotes health information behaviors through individual psychological traits. High levels of self-monitoring are associated with increased vigilance and sensitivity to self-relevant information, particularly that pertaining to health, risks, and social evaluation. A positive family atmosphere reduces individuals’ sense of phobia toward sensitive topics, thereby increasing the likelihood of active engagement with health information rather than avoidance. Furthermore, individuals with high levels of self-monitoring capacity tend to deal with health in a more in-depth manner, such as through reflection and comparison. Family discussions may stimulate initial interest. In this situation, high levels of self-monitoring drives individuals to seek information from broader sources (e.g., the internet and news) to form a clearer understanding of the relevant content. These findings are similar to the claims of information foraging theory: When families provide initial relevant information, individuals with high self-monitoring are adept at searching for high-quality information through the network of sources (58).

We also found that family functioning affects discussions about HIV/AIDS with others by promoting self-monitoring. Ecological systems theory asserts that the family, as the most proximal system to the individual, interacts with the individual and influences the person’s development (59). For example, Chu suggested that a family supports its members in two ways. First, by providing necessary information and facilities; second, by sharing existing emotions (60). Therefore, young students from well-functioning families may have acquired some degree of HIV-related knowledge through family sharing or exchange. However, in light of the influence of China’s traditionally conservative culture regarding sexuality (61), conservative attitudes persist when discussing topics such as AIDS even in well-functioning families. Individuals who exhibit high self-monitoring capacity are more sensitive to nonverbal cues, such as facial expressions and tone of voice, during interpersonal interactions. This ability enables them to seize appropriate opportunities to initiate discussions and avoid conflicts, such as choosing relaxed communication contexts. In addition, young students with sexual experience may pay more attention to sexual health, and their experience gives them stronger motivation to discuss topics related to HIV/AIDS. Awareness of health risk prompts them to actively seek family support. High levels of self-monitoring can help individuals effectively control their own emotions (e.g., embarrassment or anxiety) and recognize the emotional responses of other people. For instance, when discussing sensitive topics, communicating as a “caregiver” rather than a “preacher” can alleviate tension, and reduce the defensiveness of family members, thereby increasing the frequency of discussions about HIV/AIDS or sexually transmitted diseases with others. For young students, especially those who exhibit high levels of sensitivity, health knowledge dissemination outside the family has been proven to be effective. In summary, school or public health institutions can implement comprehensive HIV/AIDS prevention programs by integrating conventional face-to-face teaching with digital learning platforms, interactive media channels, and structured extracurricular initiatives (62).

### 4.3 Limitations

Our study has several limitations. First, the data used in this research are cross-sectional. Although the associations among family functioning, self-monitoring, and preventive behaviors were identified, causal relationships could not be conclusively established. Subsequent investigations should prioritize the acquisition of temporally sequenced datasets to elucidate potential causal mechanisms. Second, although the sample included students with sexual experience, the current investigation did not assess either the prevalence of sexual activity or the sources of sexual partners, which represent important avenues for future inquiry. Third, the relationship between family functioning and HIV/AIDS prevention behaviors may involve additional intermediary factors. Future studies can examine potential mediating mechanisms, such as social participation and partnership. Moreover, owing to economic, educational, and cultural differences across China, data from one specific city are unlikely to represent the entire population of college students nationwide. Future research should expand the sample size, such as by increasing the number of different regions or different types of universities. Researchers could also use in-depth interviews to study the relationships among family functioning, self-monitoring, and HIV/AIDS-related preventive behaviors.

## 5 Conclusion

This study revealed that family function directly affects the HIV/AIDS-related preventive behaviors of young students with sexual experience and that self-monitoring plays a mediating role in the relationship between family functioning and HIV/AIDS-related preventive behaviors. Therefore, it is necessary to add family functioning education to public health programs or develop family intervention tools. This approach could help families provide scientific and easy-to-use educational resources. This approach can improve parents’ awareness of sexual health and HIV/AIDS prevention and enable them to guide their children in developing healthy behaviors more effectively. Schools and teachers should offer sex education courses or practical HIV/AIDS preventive measures to increase self-management among young students. Furthermore, courses and practice modules should be designed to teach students self-monitoring strategies that can help enhance their awareness and regulation of high-risk behaviors.

## Data Availability

The raw data supporting the conclusions of this article will be made available by the authors, without undue reservation.

## Footnotes

1
www.wjx.cn
